# Review of Robot-Assisted HIFU Therapy

**DOI:** 10.3390/s23073707

**Published:** 2023-04-03

**Authors:** Anthony Gunderman, Rudy Montayre, Ashish Ranjan, Yue Chen

**Affiliations:** 1 Department of Biomedical Engineering, Georgia Institute of Technology, Atlanta, GA 30332, USA; 2 Department of Physiological Sciences, College of Veterinary Medicine, Oklahoma State University, Stillwater, OK 74078, USA

**Keywords:** robot-assisted HIFU, high-intensity focused ultrasound, image-guided therapy

## Abstract

This paper provides an overview of current robot-assisted high-intensity focused ultrasound (HIFU) systems for image-guided therapies. HIFU is a minimally invasive technique that relies on the thermo-mechanical effects of focused ultrasound waves to perform clinical treatments, such as tumor ablation, mild hyperthermia adjuvant to radiation or chemotherapy, vein occlusion, and many others. HIFU is typically performed under ultrasound (USgHIFU) or magnetic resonance imaging guidance (MRgHIFU), which provide intra-operative monitoring of treatment outcomes. Robot-assisted HIFU probe manipulation provides precise HIFU focal control to avoid damage to surrounding sensitive anatomy, such as blood vessels, nerve bundles, or adjacent organs. These clinical and technical benefits have promoted the rapid adoption of robot-assisted HIFU in the past several decades. This paper aims to present the recent developments of robot-assisted HIFU by summarizing the key features and clinical applications of each system. The paper concludes with a comparison and discussion of future perspectives on robot-assisted HIFU.

## 1. Introduction

High-Intensity Focused Ultrasound (HIFU) is a minimally-invasive, radiation-free technique that leverages the thermo-mechanical capabilities of ultrasonic waves for targeted treatment of malignant tumors, benign tumors (such as uterine fibroids), and other diseases (such as Parkinson’s disease) [1]. The thermo-mechanical effects of ultrasound waves were first studied on small biologic organisms by Loonis et al. in 1927 using plane waves, where rapid vibrations induced by the ultrasound waves resulted in intense heating of single cellular and small organisms [2]. However, it was not until 1942 that Lynn et al. studied the potential therapeutic capabilities of HIFU waves by investigating the impact of HIFU on in vivo brains, as well as the skin at transducer contact [3]. It was observed that by using HIFU therapies, ablation or cavitation would result in the focus of the HIFU sonication, potentially treating a controlled spot or volume [4]. In these early stages, HIFU resulted in incidental injury to the skin and subcutaneous tissue [3]. However, by the 1950s, Fry et al. demonstrated the clinical significance of HIFU using a transcranial HIFU system for creating localized lesions that treat brain tumors and Parkinson’s disease without affecting intermediate tissue [5]. Since then, HIFU has rapidly gained acceptance in the medical field as technological advances continue to improve the safety and efficacy of HIFU systems [6,7].

HIFU has several advantages over other treatment alternatives for conditions such as tumors and Parkinson’s disease [8]. In comparison to open surgery, which is often used for tumor resection, extracorporeal HIFU treatments do not require an incision, and minimal incisions are required for intracorporeal and laparoscopic-based HIFU treatments [9]. As such, HIFU avoids complications such as hemorrhaging, enables outpatient procedures without general anesthesia, and significantly reduces recovery time [10,11,12,13]. Further, most HIFU systems are equipped with control software that focuses and blocks the treatment to avoid damage to adjacent organs and tissue [12]. In comparison to radiation-based treatments, HIFU provides a safer alternative due to its radiation-free operation, enabling repeated treatment without increased risk of long-term complications from radiation [14]. In comparison to deep brain stimulation for alleviating essential tremors in Parkinson’s disease, HIFU provides a non-invasive alternative, eliminating risks associated with implanted hardware, such as unintended long-term displacement of the electrodes and recurrent surgical battery replacement [15].

While HIFU therapy has demonstrated many benefits, it is subject to several stringent requirements to ensure therapeutic efficacy and patient safety in clinical settings, all of which contribute to homogeneous focal energy delivery. For example, the target of the HIFU treatment must be within the focal depth of the HIFU transducer, which defines the workspace of the treatment based on the transducer design [16]. Additionally, HIFU requires sufficient coupling of the patient and the transducer and an unobstructed acoustic window between the transducer and the target for efficient and safe treatment [17]. Highly attenuating structures, such as muscle, provide significant design challenges, including poor efficiency of energy transfer and excessive heating in intermediate tissue [18]. Conversely, highly reflective structures, such as bone and gas, results in an acoustic impedance mismatch between the structure and the transducer, causing skin burns from the reflected ultrasound waves [12]. Further, HIFU efficacy is subject to focal positioning accuracy, with some applications requiring sub-millimeter focal accuracy (e.g., calcified aortic valves, as seen with the Valvosoft system, and the closing of varicose veins with the Sonovein System) [19,20]. These requirements have motivated physicians and engineers to incorporate novel technologies to improve treatment outcomes.

In recent years, the incorporation of image guidance using interventional ultrasound and MRI has allowed HIFU systems to expand to a variety of clinical applications, enabling proper focal placement and control [21,22]. Leveraging the real-time monitoring capabilities of these systems permits adjustments during the procedure, allowing motion compensation during respiration [23,24]. However, even with motion feedback of the target, manual adjustments are challenging for well-trained physicians; consequently, apnea induced during general anesthesia is often performed to prevent organ motion [25,26]. To mitigate these complications, robotic positioning of the transducer head, combined with image-guidance techniques, has been developed to enable high-precision, extracorporeal treatment [27]. Emerging HIFU systems utilize robots with multiple degrees of freedom (DoF) to achieve a pre-planned treatment with the system software. Within the system navigation interface, pre-operative scans and path-planning algorithms provide a treatment that avoids surrounding structures for improved safety while enabling homogeneous ablation volumes [28]. Many systems include additional feedback mechanisms to maintain appropriate acoustic coupling and thermal treatment, such as thermal feedback, reflected pressure feedback, and force feedback [29,30].

Robotic HIFU is a promising therapy for a variety of applications; however, the selection of commercially available systems is quite limited. In recent years, several unique robot-assisted systems have been developed for safe and accurate HIFU treatment, and clinical studies are being performed to test their efficacy. Due to HIFU’s non-invasive nature and success outside of oncology, novel use cases are being explored with emerging systems [31,32]. However, to date, no comprehensive review of robot-assisted HIFU and their associated imaging modalities exist. This paper provides a timely review of the emerging robot-assisted HIFU systems, discussing their competitive features and applications, image guidance and feedback mechanisms, and robotic positioning systems. Further, we seek to identify novelties provided by each system that improves localized treatment, providing an exhibition of HIFU’s ability to improve patient outcomes by reducing systemic effects. Additionally, we aim to identify the features of these systems that expand the scope of procedural feasibility for HIFU in our concluding discussion. One feature of interest to our team is motion compensation, such as respiratory motion compensation. Note that the tables in this manuscript will identify these systems. The rest of this paper is organized as follows. The review method and classification hierarchy is presented in Section 2. USgHIFU systems (Table 1), MRgHIFU systems (Table 2), and multi-modal image-guided HIFU (MMgHIFU) systems (Table 3) are discussed in Section 3, Section 4 and Section 5, respectively. In these sections, their mode of operation, key features, and applications are discussed. A general comparison of image-guided HIFU systems, as well as future challenges and directions, are presented in Section 6.

## 2. Review Method

**Figure 1:**
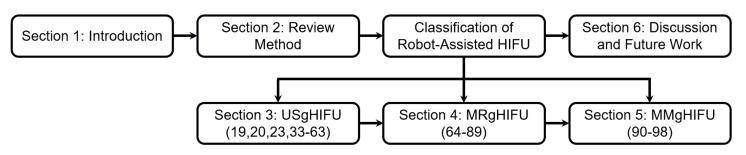
Outline of the review paper with corresponding references.

This review focuses on robot-assisted HIFU systems. This research was conducted through leading scientific research search engines and technical and medical journals, including Google Scholar, IEEE Xplore, NIH US National Library of Medicine, The International Journal of Hyperthermia, Journal of Therapeutic Ultrasound, The International Journal of Medical Robotics and Computer Assisted Surgery, and others. Initial searches were conducted in these online databases using keywords ‘‘High Intensity Focused Ultrasound OR robotic OR MR-guided OR ultrasound guided’’ to find relevant publications. The initial process yielded 112 publications. Systems that require manual positioning of the treatment system and possess no robotic positioning of the transducer are excluded from this review. The review also excludes systems that electronically steer the focus of the multi-element array without changing the position or orientation of the imaging or therapeutic probe. This resulted in a total of 70 included publications that were then reviewed and compiled. From these publications, their cited references were studied, and relevant articles were included in the final list of publications. The review of robot-assisted HIFU is classified into three primary categories: (1) ultrasound-guided HIFU (USgHIFU) systems, (2) MR-guided HIFU (MRgHIFU) systems, and (3) multi-modal image-guided HIFU (MMgHIFU) systems. The review paper structure is shown in Figure 1. The robotic positioning system, therapeutic module, and notable features for its use case are summarized for the USgHIFU systems in Table 1, the MRgHIFU systems in Table 2, and the MMgHIFU systems in Table 3.

## 3. Ultrasound-Guided HIFU Systems

### 3.1. HIFUPlex plus 1000 System

The HIFUPlex Plus 1000, developed by Verasonics, is a commercial system used for evaluating HIFU procedures on small animals. Small animal procedures are ideal for the characterization of HIFU-enabled tissue destruction, drug delivery, immunomodulation, and stem cell homing. The HIFUPlex 1000 Plus can be fitted with an annular array of up to 2.0 MHz and can achieve axial steering of a focal depth of up to 50 mm [33]. Real-time imaging is achieved with a phased array imaging transducer for 2D slices and 3D volumes [33]. The transducer positioning system includes a 2-axis linear system that permits Cartesian adjustments of up to 100 mm along both axes. The range of motion of the positioning system, combined with the electronic beam steering, ensures a large imaging/treatment area for small animal procedures [34].

One advantage of this system is the use of the concentric annular array transducer. Annular arrays enable an increased focal depth with a relatively limited number of elements in comparison to other multi-element transducers [35]. This array, coupled with the multi-focal zone positioning strategy that improves microvascular permeabilization, presents a promising novel approach for improved cancer treatment. Another advantage of this system is its modularity. The HIFUPlex Plus 1000 software allows visualization using B-mode, Doppler, and harmonic ultrasound imaging modes [33]. Additionally, the phased array imaging transducers can monitor treatment using thermal strain imaging and peak positive/negative pressure, enabling accurate heating rate prediction.

### 3.2. VIFU 2000 System

The VIFU 2000, developed by Alpinion Medical Systems, is a commercial HIFU system for extracorporeal treatments on animals up to 50 lbs. This system’s clinical applications include tumor ablation, mild hyperthermia, and immunotherapy applications. The VIFU 2000 has been used to study the effects of cavitation, as well as drug delivery in pancreatic cancer and immunotherapy applications [36]. A unique chemoimmunotherapy application of this system has been demonstrated by Ektate et al. with a triggered release of loaded temperature-sensitive liposomes, increasing the M1 macrophage expression in the presence of HIFU [37,38]. The system consists of either a 1.0 or 1.5 MHz HIFU transducer and a confocally mounted linear imaging transducer [39]. The transducers are positioned using a 3-axis robot composed of linear slides that provide a precision of 0.01 mm. This target point accuracy, coupled with the novel implementation methods listed above, can improve localized cancer treatment while mitigating severe systemic toxicity.

This system’s notable features include its modularity. The VIFU 2000 has a wet-type and dry-type configuration with separate platforms to achieve different focal depths needed for various treatments. For the dry platform, the 3D positioning system operates above the patient. The wet-type configuration is intended for smaller animals and utilizes a water tank for acoustic coupling. In this configuration, the 3D-positioning system places a small animal platform perpendicular to the ground.

### 3.3. PRO2008 System

**Figure 2:**
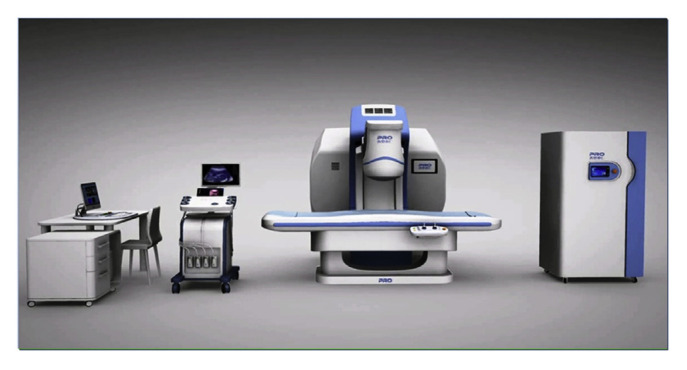
PRO2008 system developed by Shenzhen PRO HITU Medical Co., Ltd. The C-arm (**center**) positions the transducers for overhead HIFU treatment with a multi-element array [42]. Treatment is monitored by the control system (**left**), and the HIFU delivery is maintained with the water cooling system (**right**).

The PRO2008, developed by Shenzhen PRO HITU Medical Co., Ltd., (Shenzhen, China) is the first HIFU system to have Chinese Food and Drug Administration (CFDA) approval. It is a commercial, ultrasound-guided, extracorporeal HIFU system intended for treating uterine fibroids, myoma, adenomyosis, placental implantation, scar pregnancy, and others [41,42,43]. The system consists of a 1.2 MHz HIFU phased array transducer for ablation and a color Doppler imaging ultrasound transducer. These transducers are mounted to a robotically positioned C-arm that provides omni-directional rotation and axial translation to ensure optimal selection of the ultrasound treatment channel, as shown in Figure 2. For accurate positioning, the probe is also fitted with a laser light positioning system to ensure that the transducer head remains in contact with the skin.

The noteworthy features of this system include the accuracy of the HIFU treatment, real-time monitoring capability using sonographic monitoring, and integrated safety features. The C-arm positioning system and beam steering of the multi-element array enable a focal spot accuracy of 1.1 × 1.1 × 3.3 mm${}^{3}$. The sonographic monitoring provides a temperature map to control the range of ablation while also monitoring the blood flow of the surrounding areas to evaluate HIFU treatment efficacy. The treatment planning system also incorporates safety features, such as maximum treatment depth limits to avoid uterine and nerve damage. Note that the treatment planning system and the sonographic monitoring capabilities reduce the risk of uneven heating that can cause tissue damage or other adverse effects.

### 3.4. SCARA Robot-enabled HIFU

An et al. developed an extracorporeal robotic positioning system using a 4-DoF SCARA robot (YK400XG, YAMAHA, Japan) coupled with existing ultrasound imaging equipment. The system consists of a 2.0 MHz HIFU transducer for tumor treatment [44] and a portable diagnostic ultrasound system (Prosound Alpha 6, ALOKA, Japan) with color Doppler sensitivity for real-time monitoring. In this system, the robot end-effector and the ultrasound imaging probe are equipped with IR-reflective marker spheres, which are detected by an optical tracker (Northern Digital, Polaris Spectra). Using coordinate transformations, 2D slices of the tumor are collected from the ultrasound system using a motor-driven linear slide and are reconstructed into a 3D volume in the robot frame [44].

**Figure 3:**
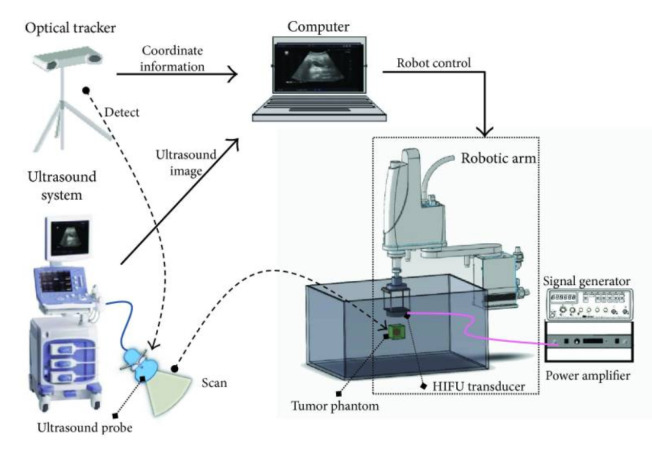
System overview of coordinate transformations between the ultrasound image and the SCARA robot frame developed by An et al. [44].

The purpose of this system was to design a low-cost, robotic system that combines existing ultrasound imaging equipment. Thus, while it lacks path planning and respiratory motion tracking, it is capable of providing a positioning error of 1.01 mm and HIFU ablation accuracy of 1.32 mm, primarily attributed to the coordinate transformation methods and calibration techniques [44]. Using optical transformation methods, online coordinate registration could be performed to ensure the HIFU focal point remains at the target in real-time (Figure 3).

### 3.5. Portable Extracorporeal HIFU

Yonetsuji et al. developed a portable extracorporeal HIFU system designed specifically for breast cancer treatment. The system consists of a 4-DoF, chain-driven serial robot enclosed in a water tank for acoustic coupling [45]. Three joints are used for planar positioning and orientation, and one joint is used for rotation about a central axis (i.e., the tumor location). This robot positions the diagnostic imaging probe and multi-element 2.0 MHz HIFU transducer using real-time monitoring. However, it should be noted that the robot end-effector and the HIFU focus are adjusted and located by visual feedback.

The most notable feature of this system is the unique rotary HIFU delivery system that moves the insonification region about a vertical axis. By adjusting the ablation pathway, skin burns can be minimized that would otherwise occur as a result of heat accumulation along the ablation path. This effectively allows the system to operate around a range center of motion (RCM) to provide a complete and homogeneous ablation of the tumor volume, improving outcomes [46]. Additionally, the compact form factor of this system allows portability by cart and can be positioned beneath a patient table for a patient in a comfortable, prone position.

### 3.6. FUSBOT

**Figure 4:**
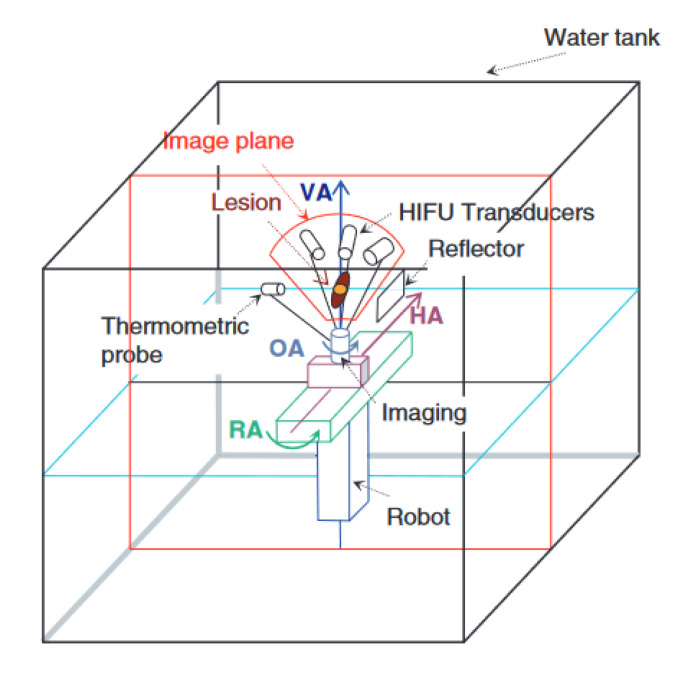
FUSBOT system with multiple transducers in a water tank. VA, HA, RA, and OA represent the vertical, horizontal, rotation, and orientation axes, respectively [47].

Chauhan et al. developed a set of robots known as the FUSBOTs (Focal Ultrasound Surgery RoBOTs) for breast-, urological-, and neuro-surgery [47]. The primary contribution of these systems is their broad scope for a variety of tumors. The robotic manipulator and kinematics are specific to their application. Both the HIFU transducers and diagnostic ultrasound transducers are positioned on the robot, as shown in Figure 4. The diagnostic imaging transducer determines the image coordinates to aid in positioning the robotic manipulator relative to the patient. Temperature readings are recorded using a thermistor sensor module, providing closed-loop feedback control of the HIFU dosage. In addition to the temperature feedback, the control algorithms can adjust the acoustic power of the HIFU transducer according to the tissue reflections. The FUSBOT control algorithms achieve a maximum positioning error within 0.5 mm. Three versions of the FUSBOT are described in this review; however, additional jig axes and interchangeable end-effectors can allow the FUSBOT to also reach trans-abdominal tumors [48].

### 3.7. FUSBOT^BS^

The FUSBOT^BS^ (Breast Surgery) consists of three 3-DoF base units that position the transducers. Two degrees change the position and orientation of the end-effector spherically, and one degree adjusts the focal depth [49]. The robot lies beneath a water tank for acoustic coupling, and the end-effector positions the HIFU probe within the water tank. At the top of the water tank is a window that the patient lies along in the prone position. This window allows the area of interest to be reached by the probe. The reported end-point accuracy of the HIFU focus is within 0.2 mm.

### 3.8. FUSBOT^US^

The FUSBOT^US^ (Urological Surgery) changes the end-effectors of the FUSBOT^BS^, allowing multi-probe and multi-route access to the tumor. This multi-probe approach achieves adequate dosage to the focal zone and low dosage exposure to areas that possess an inhibited acoustic window [47,50]. The guided user interface (GUI) of this system references online images for planning multiple routes for surgery through the trans-abdominal side of the patient for the treatment of tumors in urological organs.

### 3.9. FUSBOT^NS^

The FUSBOT^NS^ (Neuro-Surgery) delivers both single- and multi-probe treatment of targets in the brain following craniotomy. Pre-operative MRI scans calculate the 3D volume of interest. The HIFU transducer couples the *dura mater* membrane surrounding the brain and transmits the ultrasound energy through the acoustic window created by the craniotomy [48]. The HIFU transducer is actuated by the 7th-DoF of the Hexapod system of the Neurobot. The Neurobot is designed for precise drilling of the bone at the skull base with a micro-positioning parallel manipulator, enabling system accuracy within 0.1 mm [51].

### 3.10. Alpius 900 System

**Figure 5:**
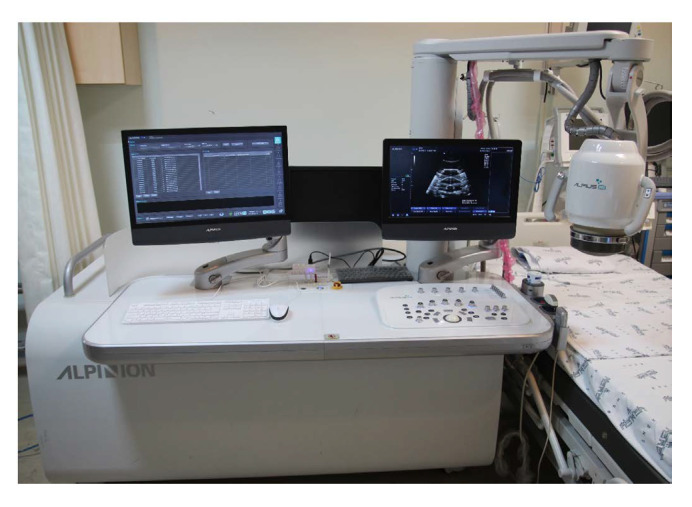
Alpius 900 system contained with the relatively small dimensions of 1.8 × 1.2 × 1.6 m${}^{3}$ for portability and streamlined workflow on a treatment table [52].

The Alpius 900 is a commercial, portable HIFU system developed by Alpinion Medical System for extracorporeal ablation and hyperthermia of stationary tumors (thyroid, breast, etc.) [52]. A 1.0 MHz multi-element HIFU transducer and imaging transducer are mounted confocally at the end of a robot hybrid positioning arm. The imaging transducer is used for treatment planning and real-time monitoring. The hybrid positioning arm rotates the imaging transducer for volume acquisition and controls the positions of the HIFU transducer. The positioning arm shown in Figure 5 is initially placed by the physician, followed by fine robotic adjustments based on ultrasound image feedback.

This system has the advantage of minimal repositioning due to the software pre-targeting tools and 3D modeling of the volumes for optimal treatment path planning [52]. During the treatment, the Alpius 900 HIFU system continuously monitors the temperature of the tissue being treated to ensure that the correct amount of energy is being delivered to the target area. This reduces heating to healthy tissue. Some notable safety features include active cooling and degassing for ensuring safe ablation temperatures and acoustic coupling with the HIFU transducer throughout the procedure. Furthermore, the portability and streamlined workflow on a treatment table enables the ease of clinical adoption.

### 3.11. 5-DoF Breast Cancer HIFU

**Figure 6:**
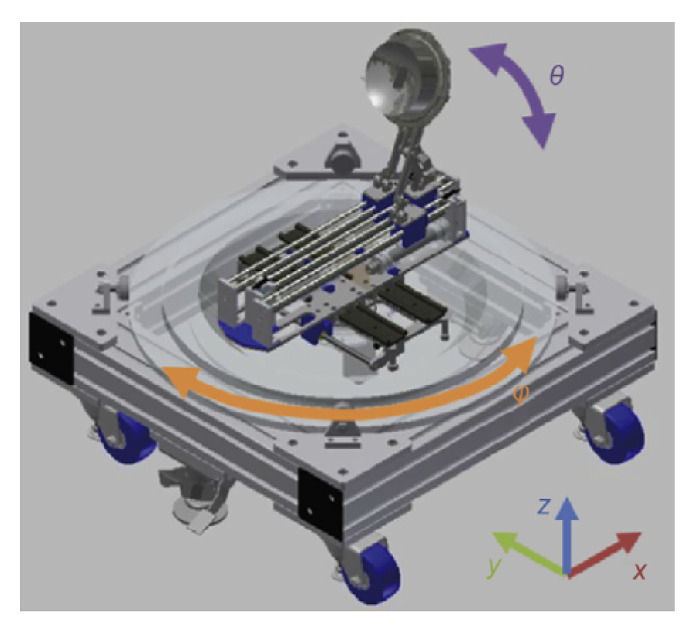
Five-DoF parallel-robot HIFU system developed by Tang et al. that can fit within a water tank beneath the patient table for breast tumor treatment [53]. The motion and axes of this positioning system are shown.

Tang et al. developed a portable HIFU system intended for the treatment of breast cancer. This system is fit with a 2D-linear imaging transducer confocally mounted to a 2.0 MHz multi-element HIFU transducer [53]. The transducers are optimally positioned and oriented using a 5-DoF parallel-link robot in a water tank beneath the patient table, as shown in Figure 6. The water tank is used for acoustic coupling. Compared to other breast cancer treatments, the patient is positioned in a more comfortable prone position, aligning the breast with the workspace of the system. Ultrasound images are used for 3D volume reconstruction, and the system software generates a path plan containing a sequence of robot poses, which are executed with the HIFU transducer driver system [53]. Within the system software, the user can select the distance and diameter of the treatment spots.

This system has several advantages, including a more homogeneous treatment area due to the orientations achieved through the parallel-link robot positioning system [53]. This system is capable of precise electrical steering of the focal point for precise HIFU ablation. The treatment software and positioning system achieves a centroid location difference of less than 2 mm and a clinically acceptable treatment area overshoot. This system possesses greater freedom of orientation with minimal repositioning, making it a highly portable option for patients.

### 3.12. Valvosoft System

Valvosoft, developed by Cardiawave, is intended to treat calcified aortic stenosis. Aortic stenosis is caused by the calcification of parts of the aortic valve that prevent the aortic valve from opening, leading to poor heart perfusion. The device consists of a high-power, multi-element transducer with a frequency of 1.25 MHz for electronic steering of the focal point and a 2D echocardiography guidance module for real-time guidance [19]. The transducers are initially positioned by the operator with real-time B-mode imaging for treatment planning. The UR-5 robot, a 5-axis motorized robotic arm, is then used to precisely position the imaging transducer for a short-axis view of the aortic valve.

The most notable feature of the Valvosoft system is its unique application enabled through the motion compensation and treatment planning in the Valvosoft software. In comparison to the standard treatments (valve replacement or balloon valvuloplasty), Valvosoft is an extracorporeal procedure without radiation that can produce satisfactory results with no adverse effects at 1-month follow-up [19]. This can significantly reduce treatment and recovery times associated with traditional valve replacement procedures.

### 3.13. UR3 Robot-Based HIFU System

**Figure 7:**
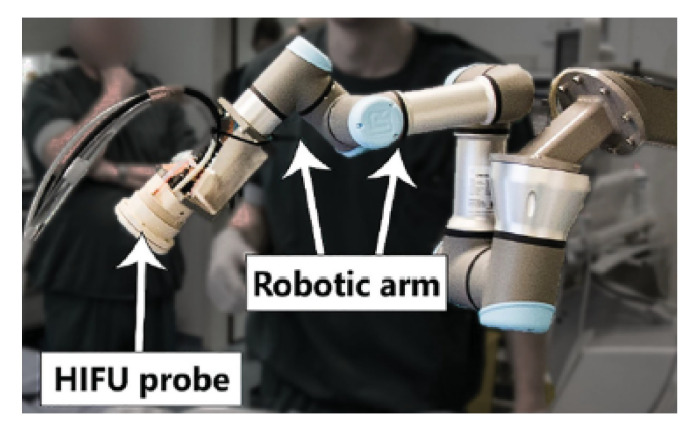
Six-DoF system developed by Groen et al. that treats atherosclerosis with precise HIFU delivery to the arterial wall of the peripheral artery [54].

Groen et al. developed a 6-DoF HIFU system intended for the treatment of atherosclerosis, shown in Figure 7 [54]. Atherosclerosis is the accumulation of plaque in the arteries, reducing blood flow and oxygen within the blood. Ablation of the arterial wall is hypothesized to promote plaque stabilization and shrinkage [55]. The system features a multi-element HIFU transducer of 3.5 MHz with a focal length of 45–55 mm [54]. Confocally mounted to the center of the imaging probe is the imaging transducer, enabling real-time-target selection using B-mode images. These transducers are positioned by the 6-DoF UR3 robot arm (Universal Robots, Odense, Denmark), providing a targeting accuracy of approximately 1 mm.

The advantages of this system include the closed-loop HIFU energy delivery modulation and the precise positioning of the focus with electronic steering and positioning of the 6-DoF robot arm. Additionally, the control software and embedded safety features ensure patient safety during the therapy of arterial diseases. For example, the feedback mechanisms used for procedural monitoring rely on monitoring echogenicity, which increases with rising temperature. As a result, the software is capable of measuring rapid increases in echogenicity and temperature and can halt the procedure to avoid skin burns and reduce damage to patients.

### 3.14. JC200 System

**Figure 8:**
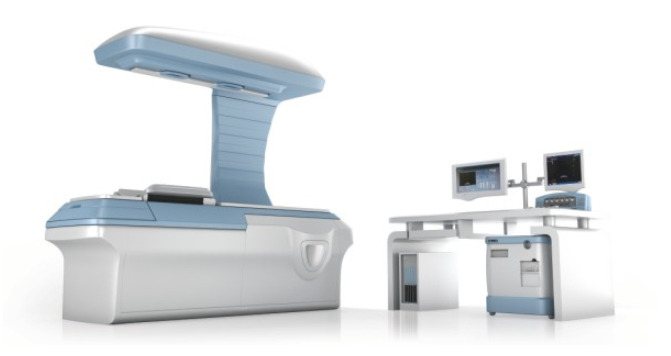
JC200 System with a 6-DoF motion system connected to the patient table for overhead treatment [58].

The JC200 is a commercial system developed by Haifu Medical. It is intended for the treatment of benign gynecologic tumors (e.g., uterine fibroids). Uterine fibroids are amongst the most common tumors for women of reproductive age and can reduce fertility [56]. Treatment of uterine fibroids and other tumors with HIFU serves as a non-invasive alternative to laparoscopic procedures or surgeries. This system has also been used for the treatment of the liver, bone, breast, and pancreas. However, it should be noted that to reduce the respiratory motion of organs, anesthetic techniques are used instead of robotic techniques [57]. The JC200 is fit with a single-element HIFU transducer with a central frequency between 0.8 and 2.4 MHz. The transducer module is positioned overhead with a 6-DoF motion device for precise HIFU treatment [58]. The positioning system and transducers are fit to a treatment table, as shown in Figure 8, to achieve a maximum range of 120 mm in the latitudinal and longitudinal directions and 180 mm in the elevation direction [58]. The JC200 achieves a focal spot of 1.1 × 1.1 × 3.3 mm${}^{3}$ with a focal positioning accuracy of ±0.1 mm. The treatment planning and real-time monitoring of the treatment are visualized using diagnostic ultrasound transducers that construct a 3D volume in the system control software [59]. This system is reported to have a mean fractional ablation (non-perfused volume/nodule volume of tumor) of 94% for tumors ranging from 0.9 to 2.1 cm${}^{3}$ [59].

The primary advantages of the JC200 are its large range of motion and accurate focal precision, capable of a 10-cell wide margin between ablated and unablated tissue [59]. The capabilities of the JC200 are further expanded by the interchangeable transducers and control software. The software’s ability to provide real-time dosage adjustments based on ultrasound image feedback makes this system clinically relevant for patient use.

### 3.15. 6-DoF HIFU with Motion Compensation

**Figure 9:**
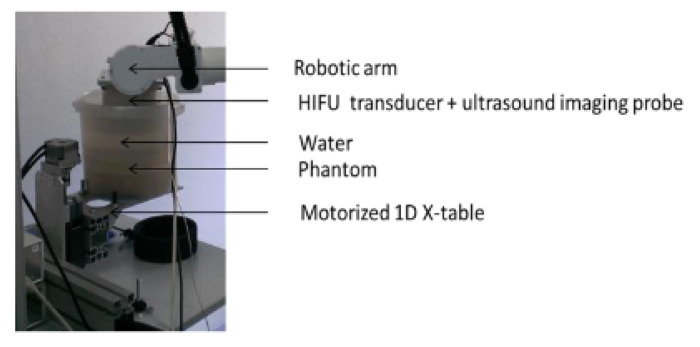
Six-DoF motion-compensated HIFU system developed by Chanel et al. [23]. Motion compensation is enabled through ultrasound motion estimation using speckle tracking.

Chanel et al. developed a 6-DoF HIFU system with active motion compensation intended for the treatment of tumors in the abdomen. The active motion compensation is based on real-time ultrasound imaging feedback and motion estimation [23]. The system features a 2.9 MHz HIFU transducer and a confocally mounted phased array ultrasound probe. These transducers are positioned by the 6-DoF robotic arm (Sinters, France), which positions the treatment focus using reconstructed B-mode images that enable motion estimation utilizing speckle tracking (Figure 9). Following the motion estimation, the system follows a position-based visual surveying scheme using a trajectory generator and PID controller to regulate the joint input velocity [23].

The primary advantage of this system is the real-time motion compensation algorithm, which is relevant for clinical applications. The 6-DoF arm and target estimation maintain target tracking of the focus location to reduce the tracking error by 80% (<0.88 mm). Thus, this system could significantly reduce treatment times normally expected in traditional breath-holding techniques (4.5–7 h).

### 3.16. FUTURA System

FUTURA is a collaborative project developed by a consortium of research groups. It consists of a 6-DoF robotic USgHIFU system for various tumor treatments. The system is fit with two identical 6-DoF robot manipulators (IRB-120, ABB, Zürich, Switzerland) used to position the 3D ultrasound probe and the single-element HIFU transducer above the patient platform. Each robot arm is fit with a 6-DoF force and torque sensor at the end-effector. The FUTURA is also fit with an optical sensor on the robot arm, which calibrates the end-effector using the ultrasound images for accurate spatial information of the target. This produces an ultrasound reprojection error of 0.42±0.28 mm [60].

A primary advantage of this system is the closed-loop feedback algorithm used to improve ablation volume accuracy. The FUTURA system software identifies the target with a semi-automatic segmentation algorithm of the acquired ultrasound volumes. This algorithm extracts the center of mass from both the initial segmented volume and the lesion to provide online monitoring of the ablation accuracy [60]. The position errors between the centers of mass of the target and of the lesion are calculated and adjusted, providing a targeting error of 0.66±0.19 mm and 0.66±0.34 mm error in the radial directions and 3.67±1.51 mm error in the axial direction of the transducer after the initial sonication. Note that the targeting accuracy improves with each iteration of sonication.

### 3.17. Sonovein HIFU System

**Figure 10:**
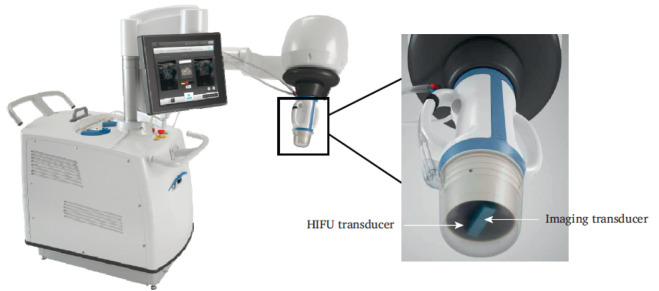
Sonovein system, developed by Theraclion, is intended for the treatment of varicose veins with sub-millimeter precision [20].

The Sonovein system, developed by Theraclion, is intended to treat varicose veins with HIFU by shrinking the vein and reducing variable blood flow. Varicose veins are enlarged veins of the leg that can significantly influence the flow of blood to the heart, causing extremity weakness, heaviness, pain, and clotting. HIFU is an appealing non-invasive option to treat varicose veins, providing an outpatient procedure under local anesthetic [62]. The 3.0 MHz treatment head of the Sonovein system is equipped with a cooling and coupling kit and includes a linear ultrasound probe that is confocally mounted, as shown in Figure 10 [20]. Initial positioning is performed manually by the physician and ultrasound imaging is used for vein localization. Following identification, Sonovein’s Matlab-based software automatically adjusts the power delivered in each pulse with the depth of the vein based on ultrasound image feedback [62]. The robotic positioning system precisely moves toward target areas and treatment sites selected by the physician. The robotic positioning system allows for treatment 2 mm away from the femoral junction with a focal spot width of 0.5 mm [20].

The primary novel contribution of this system is its unique application. Compared to traditional modes of treating varicose veins, the treatment time is reduced, and local anesthetic is not necessary, reducing complication risks. Currently, clinical trials are underway to assess Sonovein’s ability to shrink varicose veins and restore normal blood flow [63]. Early clinical data suggest that this approach may improve outcomes in patients.

## 4. MRI-Guided HIFU Systems

### 4.1. Medsonic System

**Figure 11:**
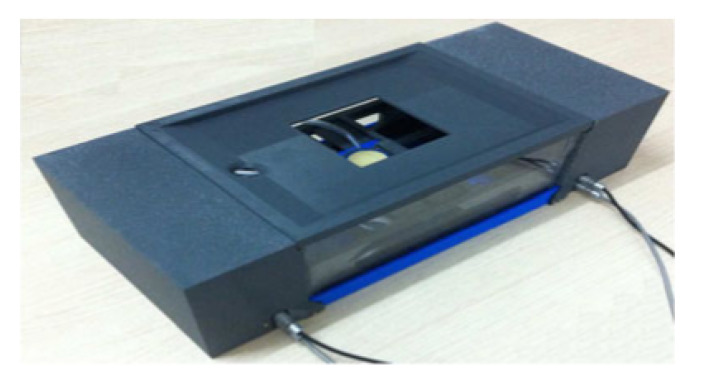
Medsonic system developed for small animal studies [64]. Acoustic coupling relies on the submersion of the treatment region of the animal inside the water basin.

The MRI-guided HIFU system for small animal experiments developed by Medsonic Ltd. is a 2-DoF system intended for ablation and other HIFU procedures. The system has a compact form factor with a height of 14 cm, a length of 45 cm, and a width of 25 cm, allowing it to sit on the MRI patient table. The system has a relatively large range of 20 cm in the *X*-direction and 8 cm in the *Y*-direction to position the HIFU transducer. It is fit with a single-element transducer that is positioned by piezoelectric motors toward the acoustic window, as shown in Figure 11. For appropriate coupling and cooling, the transducer is immersed in the centrally located water tank. A small animal may be placed in the supine or prone position with the treatment area submerged. The system’s software supports MR-thermometry for treatment monitoring, transducer coordinate control, and MR-compatible camera control for procedural feedback and monitoring.

The advantages of this system include its low cost, simplicity, and open-access presentation (CAD available online). The use of a fixed focal depth transducer enables simple registration and motion planning for evaluating HIFU applications in small animal studies [64]. Additionally, Yiannakou et al. reported that a potential conversion of this system for human treatment tables could extend the range in the *X*- and *Y*-directions to 30 and 9 cm, respectively, for treatment of the human liver, kidney, brain, fibroids, bone, and breast tumors [64].

### 4.2. 4-DoF MR-conditional HIFU System

**Figure 12:**
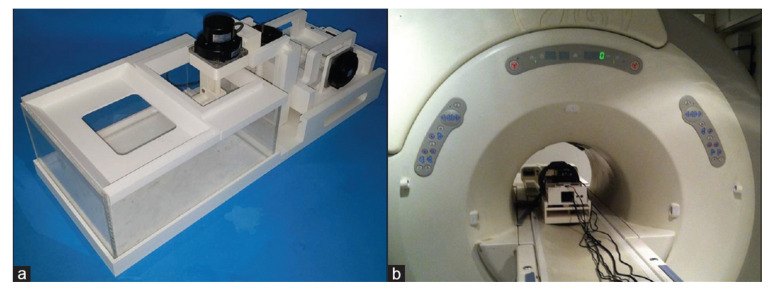
(**a**) MRI-guided system for focused ultrasound ablation in companion animals by Damianou et al. [65]. (**b**) Device located at the isocenter of the MRI scanner.

Damianou et al. developed a robotic system for MR-guided ablation proposed for clinical trials in companion animals. This robotic system consists of 3D-printed ABS parts and has a compact form to fit in any commercial 1.5T and 3T MRI scanner. This system has 4-DoF consisting of three linear axes and one angular axis. The axes position the HIFU transducer within the water tank while the companion animal lies above the transducer. The axes are driven with piezoelectric ultrasonic motors that are placed outside the water container, as shown in Figure 12 [65]. This device is fitted with a single-element transducer that can achieve a variety of orientations to treat multiple organs. The positioning device has a range of 5 cm on the *Z*-axis and 7 cm on both the *X*- and *Y*-axes. The transducer can rotate ±90°. The optical encoders allow for a linear axis motion error of 0.06 mm and a 0.05° error for the angular axis. This device’s range of motion allows the patient to be treated in the prone position for possible treatment of the breast, liver, kidney, and pancreas. This device may also be used for bone palliation in the supine position.

The main innovation of this robotic system is the electronic actuator placement, which is outside of the water container. The transducer is attached to an angular stage that submerges the transducer in the water basin for acoustic coupling for homogeneous energy delivery. This angular stage reduces the complexity of passing the transducer arm through water-proof mechanisms. Notable features of this device include a user-friendly interface that allows for manual control of the robotic device motion, control of the focused ultrasound amplifier and streamlined communication with the MRI scanner. This system software includes temperature monitoring through MR-thermometry and the ability to obtain camera images with an MR-conditional camera.

### 4.3. 4-DoF HIFU System for Bone Metastases

**Figure 13:**
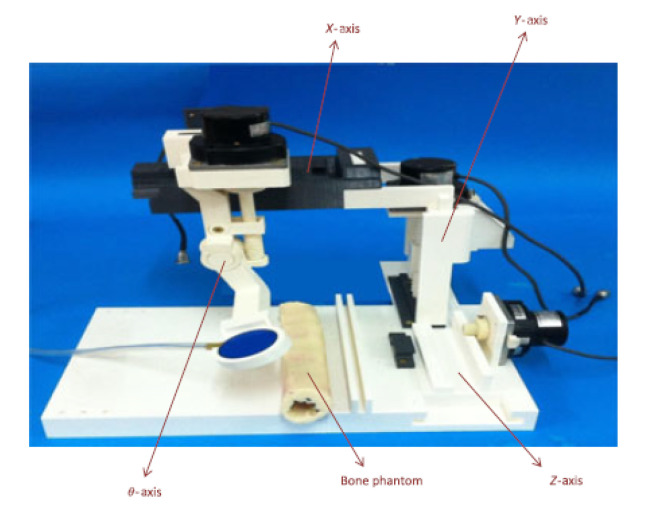
MRI-guided focused ultrasound robotic system developed by Menikou et al. for HIFU treatment of small animals [66].

Menikou et al. developed a 4-DoF robotic system for the HIFU treatment of bone metastases pain palliation [66]. Bone metastases and periosteal nerve endings can be ablated with HIFU [67]. Due to the high attenuation of bone, HIFU wave penetration is reduced. However, the ablation of nearby periosteal nerves can significantly reduce patient pain [68]. This system, shown in Figure 13, fits within the MRI bore and has a range of motion of 8 cm in the *X*-, *Y*-, and *Z*-axes and a ±90° axis of rotation. The axes are driven by piezoelectric ultrasonic motors. This lightweight and portable system sits on the treatment bed of the MRI machine, and the robotic system positions the therapeutic transducer above the region of interest.

Notable features of this system include its multi-path treatment options. This system’s software has six possible algorithms that provide a path plan for treatment with the robot, enabling optimal treatment selection in a large workspace [69]. Further, this can reduce treatment time as repeated manual robot positioning may not be necessary. This system allows the user to choose the position along the angular axis, followed by a user-selected *Z*-axis position. A programmed ablation along the X-Y plane is performed according to the treatment plan algorithm. As a final note, this robot configuration enables patient placement in the supine position as opposed to the prone position. This feature, paired with its chilled water acoustic coupling that prevents skin burns, can reduce patient pain and suffering. As a result, anesthetic may be unnecessary [66].

### 4.4. 3-DoF MR-conditional HIFU System

**Figure 14:**
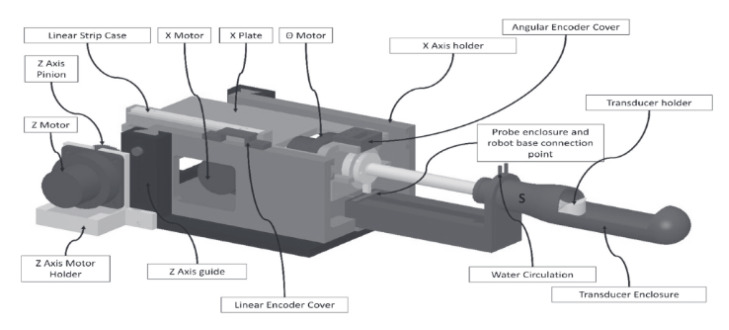
Transducer positioning system by Yiallouras et al. for transrectal treatment of prostate diseases [70].

Yiallouras et al. developed a three-axis MR-conditional robotic system for transrectal HIFU treatment of prostate diseases, as shown in Figure 14 [70]. This system includes two linear stages and one angular stage. For this system, the patient lies prone on the table with slightly elevated legs. This robot’s range of motion is 10 cm in the longitudinal direction and 7 cm in elevation, with a ±90° rotation about the longitudinal direction. The encoders for the axes allow for a measurement error of approximately 20 μm for the linear axes and 0.11° for the angular axis. A water enclosure attached to the base provides water circulation through the transducer enclosure for cooling and coupling. The spherical 3 cm diameter HIFU transducer has a frequency of 3.0 MHz and a focal length of 4 cm. This system currently uses a lumbar spine coil for MR imaging.

An advantage of this system is the control system. The system’s software allows the user to control the three axes in either user-defined steps or in grid sequences with a user-defined path. The software also logs the transducer coordinates, history of functions, images of the MR-compatible camera, and temperature measurements of the system, creating a comprehensive history of the patient’s treatment. An additional advantage of this system is the rotation of the transducer. This enables the ability to apply homogeneous energy delivery with the increased workspace.

### 4.5. 2-DoF HIFU System for Gynecological Tumors

**Figure 15:**
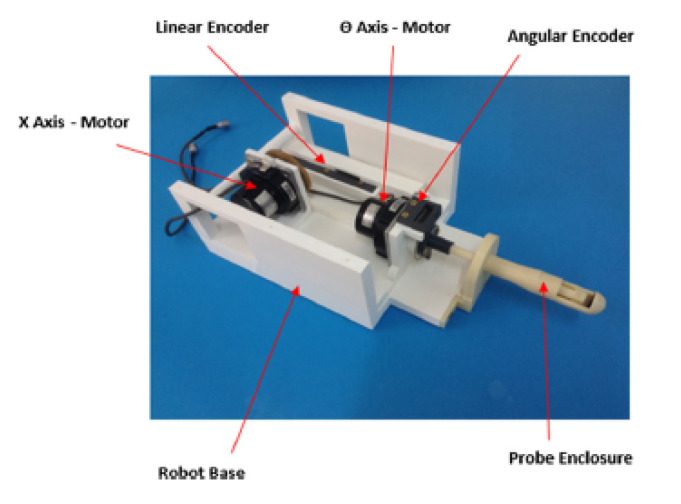
System by Epaminonda et al. for the treatment of gynecologic tumors [71].

Epaminonda et al. developed a system intended for the treatment of gynecological tumors. The positioning system is composed of only MRI-compatible materials, and the actuators are driven by piezoelectric ultrasonic motors. The components are 3D-printed with ABS plastic and maintain a compact form factor for placement on the MRI patient table. The robot is equipped with a 3.0 MHz HIFU transducer that is positioned using a linear axis with 100 mm of translation and an angular axis with ±90° of rotation, as shown in Figure 15. The transducer probe robotic-positioning system has an appropriate range of motion for treating most reported sizes of gynecological tumors. The probe is connected to a water tank for appropriate cooling and coupling.

Notable features of this system include the compact form factor. Similar to the previous system, the rotation of the transducer provides a large workspace for homogeneous energy delivery. This system also has increased image quality due to the probe-fitted imaging coil. As the imaging probe is near the lesion and far away from the piezoelectric motors, the imaging quality is minimally affected [71].

### 4.6. TRANS-FUSIMO

The TRANS-FUSIMO treatment system (TTS) is intended for the focused ultrasound treatment of upper abdominal organs such as the liver [72]. HIFU treatment of upper abdominal areas can be challenging due to respiration motion and the presence of occluding structures (e.g., ribcage) that attenuates and reflects the ultrasound energy. The TTS monitors the patient’s respiratory pattern and sonication via the MRI to electronically steer the focus at a moving target. This robotic system is fixed to the patient table and is located directly above the patient.

Notable features of this system include a thermometry temperature map, electronic blinding and beam-shaping technology for the continuous application of HIFU during respiratory motion, and an obstacle avoidance algorithm (i.e., ribs and other structures surrounding the target tissue) [73]. Each of these features aids in creating a comprehensive system that can avoid unintended application of HIFU waves, avoid skin burns from the reflection of waves off of nearby structures, and enable intraprocedural modifications for efficient HIFU ablation [74].

### 4.7. 4-DoF HIFU Robotic System

**Figure 16:**
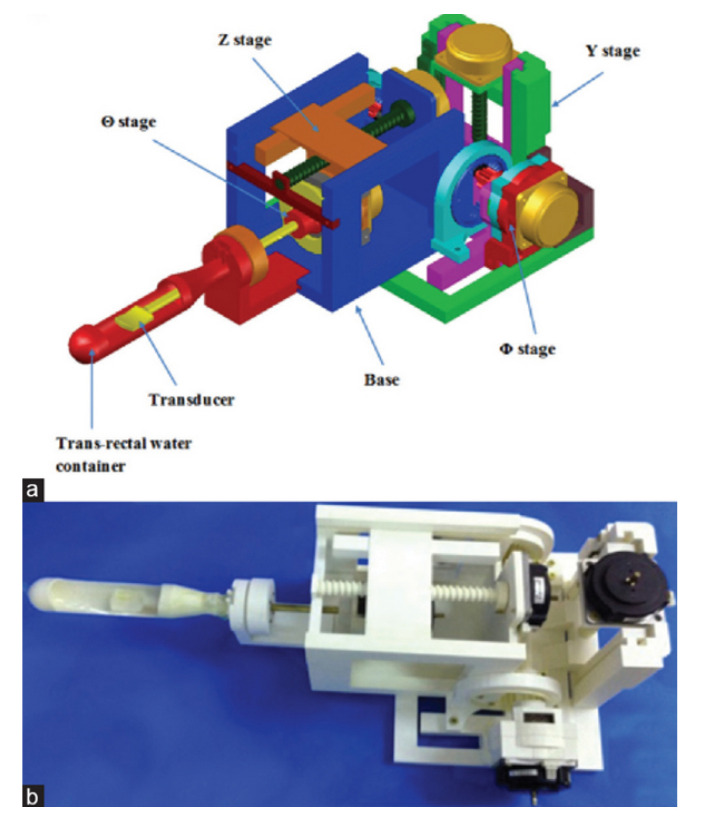
(**a**) CAD drawing of Giannakou et al.’s system for prostate cancer [76]. (**b**) Physical system with four computer-controlled stages.

Giannakou et al. developed a system for the endorectal treatment of prostate cancer. The system is equipped with a 3.0 MHz transducer with a focal length of 40 mm. This range is sufficient to treat any sized prostate after endorectal placement. The device has four robotically controlled axes, corresponding to the linear *Z*- and *Y*-axes and rotational axes about the θ and ϕ directions, as shown in Figure 16. Along the *Z*-axis of the MRI scanner, the device linearly moves the axis into the rectum to position the transducer near the prostate gland. Along the *Y*-axis of the MRI is a linear axis to change the robot elevation. The ϕ-axis aligns the endorectal probe with the rectum incline. Within the probe, the θ-axis rotates the transducer within the probe for an appropriate orientation toward the prostate gland [76]. The range of motion of the robot is 8 cm along the *Z*-axis, ±90° about the θ direction, 6 cm along the *Y*-axis, and 0°–90° about the ϕ direction. The MR-conditional encoders allow the positional measurements of the linear stage of 0.05 mm and the angular stage of 0.2°.

Notable features of this system include the small form factor, which enables placement between the legs of a patient lying in the supine position. This device utilizes thermocouples and MR-thermometry for temperature measurements in the region of interest for thermal feedback of the prostate tumor treatment. Further, in comparison to other endorectal coupling devices, this system utilizes a robotically controlled axis of a single-element transducer rather than a multi-element transducer, achieving a small footprint and allowing it to sit on the patient’s table and between the legs of the patient.

### 4.8. ExAblate 2000 System

**Figure 17:**
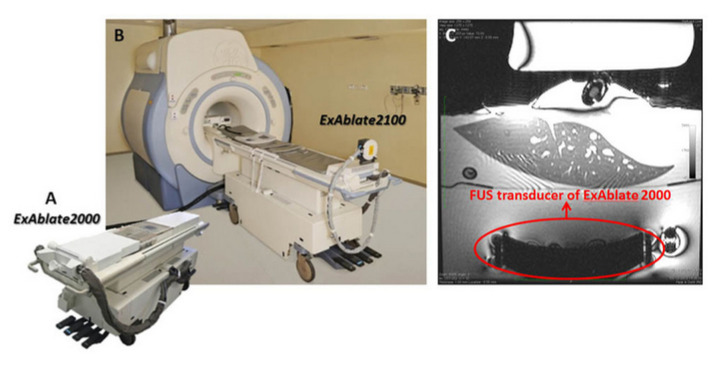
(**A**) ExAblate 2000 System, (**B**) ExAblate 2100, (**C**) ExAblate 2000 FUS Transducer in an MR Image [82].

In 2004, the ExAblate 2000 became the first commercially available MRgHIFU system. The ExAblate 2000 was developed by InSightec for HIFU ablation of tumors [77]. This device has mainly been used for treating uterine fibroids [78]. The ExAblate system 2000 has also been used for the ablation of breast, liver, and prostate cancers, as well as for triggering drug activation in both 1.5T and 3T MRI machines [79,80,81]. The system consists of a 5-DoF robot driven by piezoelectric motors to control the roll and pitch of the transducer, as well as translations in the latitudinal and longitudinal directions. The HIFU transducer positioning system resides within the patient table, as shown in Figure 17 [82]. The transducer resides in a water tank for appropriate cooling and coupling. The ExAblate system ablates volumes with a point-by-point sonication approach, with a cooling time required between individual sonications.

This system includes several notable features. The accompanying system software allows user control of the focal spot size, location, angle, and energy deposition. This enables tuning for optimal energy delivery and avoiding healthy structures. The feedback system provides real-time MR-thermometry for a spatiotemporally controlled temperature profile that is overlaid over the MR images [83]. Future iterations of the ExAblate 2000 allows for elements of the transducers to be turned off for beam shaping and beam steering [84]. It should be noted that respiratory motion is not compensated by the robotic system; rather, anesthesia is used.

### 4.9. Sonalleve System

**Figure 18:**
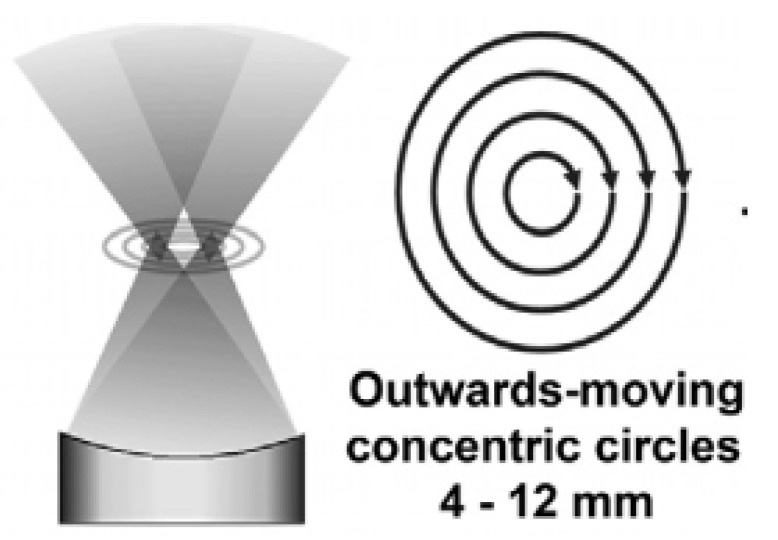
Path of electronically steered HIFU transducer focal point for Ablation with the Sonalleve System, enabling more homogeneous energy delivery [86].

The Sonalleve MR-HIFU system, developed by Phillips and Profound Medical Inc., is a HIFU positioning system that easily mounts to existing patient tables of MRI scanners. This system is aimed at treating uterine fibroids [85]. This system contains a phased array transducer for electronic and mechanical steering of the focus [86]. Volumetric ablation is achieved through electrical steering of the HIFU transducer focus in a continuous manner with concentric circular trajectories of increasing size, as shown in Figure 18. The system allows the online acquisition of thermal information to control the sonication with a thermal feedback method defined by Kohler et al. [87]. This feedback method stops the sonication when the thermal ablation and temperature profiles match the user-selected inputs. The feedback systems avoid over-ablation and damage to surrounding structures while achieving a homogenous ablation.

The notable feature of this system is the circular treatment pattern, which enables a more energy-efficient application of energy deposition during the sonication at the inner part of the trajectory [88]. This technique is also used for more efficient mild hyperthermia. In comparison to other point-by-point treatment trajectories, such as the ExAblate 2000, the volumetric ablation technique controls the heating for a larger volume per sonication. This results in shorter treatment times in comparison to other HIFU systems. The automatic adjustment of the ablation per spot due to the feedback system also decreases treatment time and increases patient safety.

### 4.10. WPI Robotic HIFU System

**Figure 19:**
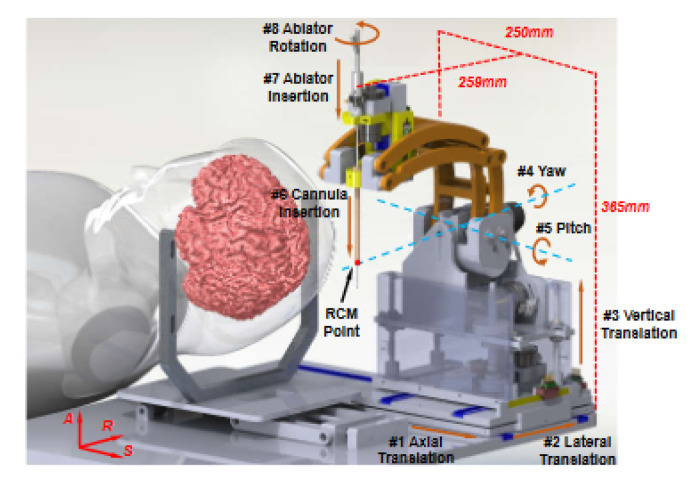
Li et al. 8-DoF system for brain tumor ablation using MR-tracking coils [89].

Li et al. developed a system for the precise ablation of brain tumors. In order to avoid the attenuation of the skull, a fixed hole in the skull is made prior to the ablation process. A needle-based HIFU applicator is positioned with an 8-DoF robot that operates within the bore of the MRI machine. Five of the robot DoF position the applicator similar to a stereotactic frame, as shown in Figure 19. The remaining three DoF insert the needle and change the orientation of the needle. The insertion module includes a plastic implant catheter that is appropriate for an ablation device of this form [89]. Along the shaft of the transducer, active tracking coils provide high-accuracy localization and orientation of the catheter. The robot is composed of 3D-printed ABS plastic and is driven by piezoelectric actuators that produce minimal noise and high accuracy. This system was validated for thermal ablation in ex vivo tissue with the tubular transducer as an active element on a fixed lamb head. Accuracy for the position and orientation of 0.50 mm and 2.00° were achieved for the needle placement.

A notable advantage of this system is the use of active tracking coils for the accurate task-space placement of the HIFU system. MR-tracking coils are small, radio-frequency coils that can be integrated with interventional devices for high-resolution localization in MRI [99,100,101,102]. Li et al. designed two custom coils to obtain ablator position and orientation with a root mean square (RMS) error of less than 1.1 mm and orientation RMS errors of less than 2.3°. This enables efficient device tracking, eliminating the need for long, high-resolution MR-imaging sequences for obtaining device position and orientation. Additionally, this ensures patient safety within the high-risk intracranial region.

## 5. Multi-Modal Image-Guided HIFU Therapy

### 5.1. Focal One System

The Focal One from Edap TMS is a HIFU system intended for the ablation of tumors in the prostate. The Focal One combines three different imaging techniques for identifying the treatment area. MR images or 3D biopsies collected prior to treatments may be overlayed on intraprocedural ultrasound images. These overlayed volumes are fitted to the ultrasound image with the system’s elastic 3D deformation algorithm called HIFusion [90]. The pre-operative 3D biopsy data may be taken from transrectal or transperineal devices, and the tumor contours are displayed on the real-time ultrasound image during treatment. The transducer and positioning system are built into the central console table for a streamlined workflow. This system contains a 3.0 MHz HIFU transducer, with a 7.5 MHz imaging transducer confocally mounted to the center of the HIFU transducer. The probe containing these transducers is controlled by a robotic system with three translation stages and one rotation stage along the length of the probe. The HIFU transducer is electronically controlled for dynamic focusing on reaching several focal depths along the tumor.

The notable feature of this system includes the multi-modal image guidance, including MR, ultrasound, and 3D biopsy data, with the multi-element beam steering for precise treatment. The system interface allows for adjustment of the ablation planning path in real time. Rosenhammer et al. report a 10 mm safety margin around the ablation zone after the HIFUsion overlay [90].

### 5.2. Innomotion Robotic System

**Figure 20:**
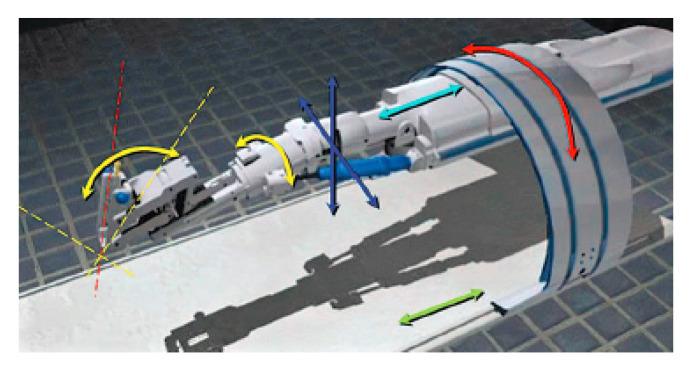
Innomotion system and its corresponding illustrated joint motions [92].

Innomotion by Melzer et al. is a pneumatically driven five degree of freedom system. The robot arm is attached to a 180° orbital ring surrounding the patient table, which has two manual adjustments for an additional 2-DoF before the procedure, as shown in Figure 20. The patient table and orbital ring structure are designed to fit within MRI and CT platforms. The robot arm may be manually positioned in the orbit region such that it can reach a patient’s lateral regions as well as operate above the patient. This allows for treatment of the region of interest in the spine, liver, kidney, breast, and other organs [92]. Innomotion can be used for a variety of applications with the following robotic positioning system and accompanying software. The system is driven by pneumatic cylinders, and the positioning measurements are achieved through fiber optic limit switches, rotational encoders, and linear encoders. The end-effector of this robot may be fitted with an adapter to hold the single-element HIFU transducer and a confocal wireless ultrasound imaging probe mounted in the center of the HIFU transducer [93].

The notable features of this system include the orbital positioning system and safety feature-rich software, enabling safe ablation across a large workspace with multi-modal image guidance. These include pressure and voltage monitoring, monitoring of the encoders, axis monitoring, and others. The transformations between the MRI scan, the B-mode ultrasound image, and the robot and tool module are calculated automatically within the control module software. This system also features telecommunication, with force feedback at the end-effector. This tele-medicine approach may be further developed for MRI-guided surgical applications.

### 5.3. Ablatherm System

Ablatherm is one of the earliest commercially available systems for prostate cancer treatment. This system has a robotic manipulator built into the patient table. With the patient lying on their side, the 6-DoF robot positions the probe adjacent to the prostate. The probe contains the HIFU transducer and the confocally mounted ultrasound imaging transducer [94]. Ablatherm fusion combines pre-treatment MR images and 3D biopsy maps with software to match the 3D biopsy volume to the ultrasound images. During treatment, the MR target and tumor locations are displayed on the ultrasound image in real time. Notable features of this system include its non-invasive nature and its simplicity.

### 5.4. Sonablate 500 System

Often compared to Ablatherm is the Sonablate, produced by Sonacare Medical [96]. Similar to the Ablatherm, this system also displays the MRI image contours of the volume onto the real-time ultrasound images. Sonablate has a two-sided transducer that allows ablation at 3 cm and 4 cm focal distance. The probe also contains a confocal imaging transducer in the center of the HIFU transducer. This system positions the imaging and HIFU transducer within the probe. The transducer may be moved along the length of the probe and rotated within the probe to provide ablation at different angles. The HIFU probe is secured and positioned prior to the ablation sequence with an articulating probe arm.

Notable features of this system include the path-planning capabilities of the probe. This path is planned using the pre-surgery ultrasound image scans from the confocally situated imaging probe along the entire prostate in both the sagittal and transverse planes. The path of the ablation treatment avoids sensitive structures such as the seminal vesicles, the rectal wall, and the prostate capsule. The Sonablate 500 monitors tissue destruction through a comparison of radiofrequency ultrasound pulse-echo signals (backscattering) at each ablation site. The backscattered echo signals provide phase and amplitude information that is used for quantifying the energy increase reflected by the induced ablation dose to the tissue in real-time [97].

## 6. Discussion and Future Work of Image-Guided HIFU Systems

Due to the significant advantages of extracorporeal treatments, HIFU has gained significant popularity, enabling its use in several different clinical applications. These include: (i) treatment of tumors of the liver, kidney, brain, bone, prostate, breast, uterine fibroids, and more, (ii) aortic stenosis [19], (iii) varicose veins [20], (iv) essential tremor in Parkinson’s disease [5], and more. These treatments often enable outpatient procedures without the administration of general anesthesia [26], reducing complications and the risk of infection associated with hospital stays. Currently, there exist several robot-assisted HIFU therapies, though there are only a limited number of commercially available systems. Initial robotic HIFU systems typically had limited degrees of freedom (i.e., 1 or 2) and fixed focal depth, such as the Ablatherm system [94]. Thus, their applications were limited to biological systems not subject to significant obstructing structures (i.e., endorectal prostate treatment). However, advancements in robotic technology have enabled significant improvements in treatment efficacy. These include multi-degree of freedom systems that enable homogeneous ablation volumes, systems equipped with end-effector force-torque feedback for proper acoustic coupling, thermometry of the treated region for treatment evaluation, cavitation prevention feedback, and electronically steered and blocked HIFU waves for obstacle avoidance.

The most prominent image-guidance methods for robot-assisted HIFU are diagnostic ultrasound and MRI. Ultrasound-guided systems rely on B-mode images for real-time monitoring of the treatment. These diagnostic B-mode imaging systems are widely available and inexpensive but require 3D-probes or additional software to reconstruct 2D slices into 3D volumes. Like HIFU transducers, imaging transducers come in various form factors and frequencies. However, the HIFU and imaging transducers must be of the same acoustic impedance and meet acoustic coupling requirements for proper treatment [4,103]. Due to these few constraints, ultrasound imaging systems are often designed to be portable, enabling prompt adoption. However, it should be noted that while temperature monitoring methods exist for ultrasounds, this feature is not available in many of the commercially available diagnostic ultrasound imaging systems, as many filtering techniques are required for a clear contrast image [104,105]. This lacking feature must be incorporated by the developers of the positioning system with access to the diagnostic ultrasound system’s software.

In comparison to diagnostic ultrasound, MRI provides high-contrast images that do not rely on an additional coupling medium. Additionally, MR-thermometry is commonly incorporated into nearly every MR-guided system, as there exist many temperature-sensitive parameters that must be monitored. However, there are several constraints that limit MRgHIFU adoption. These include the strong, applied magnetic field, the space restrictions of the MRI bore, high operation cost, and lack of portability. To accommodate the magnetic field, robotic positioning systems and the HIFU transducer used under MR guidance must be composed of components that are either MR-conditional or are appropriately shielded to prevent SNR reduction [106,107,108]. This may be accomplished with piezoelectric or pneumatic elements for positioning actuation, along with plastic components for the chassis [93,107,109]. Due to the restrictive nature of the MRI bore, the robotic positioning system must have a small footprint to fit in the MRI bore with the patient. Consequently, these systems often have a limited workspace that precludes homogeneous ablation volumes seen in USgHIFU.

In an effort to combine the advantages of real-time ultrasound imaging and the temperature monitoring capabilities of MR images, multi-modal image-guided systems have been used, such as Innomotion. By using wireless confocal imaging probes to prevent adverse interactions with the magnetic field, thermal and volumetric imaging is improved [92]. Conversely, systems such as Focal One have reported extrapolating the contours of pre-operative MR scans and overlaying this on the ultrasound image for improved boundary identification of the treatment volume [90]. Although these systems can improve procedural efficacy, it comes with higher costs and a lack of portability.

There are several different elements that result in effective HIFU treatment, all of which contribute to improving homogeneous energy delivery and reducing potential side effects. These include automatic adjustment of the acoustic power, real-time imaging, force and temperature monitoring software, electronic element steering and blocking, etc. These elements have resulted in the rapid adoption of HIFU therapies in lieu of more invasive approaches. Although many of the presented systems in this review contain these necessary elements, it is the authors’ opinion that the future efficacy of HIFU lies in the development of respiratory motion-compensated systems to expand the scope of applicable procedures and reduce the potential risk of healthy tissue damage during respiration. This is highlighted by the procedural scope of current systems. Of the 28 systems presented, only 8 provide the capability for treating organs with respiratory motion (such as the liver and pancreas [110]). Further, only five provide active motion compensation [19,23,61,73,92]. The other three rely on apnea induced during general anesthesia, which is associated with long procedural times and additional complications [111,112]. Consequently, despite the advanced path-planning capabilities of many of these systems [52,53,96,97], such as the Alpius 900 system, they are not suitable for dynamically moving organs. As a result, these systems are often limited to organs such as the prostate, thyroid, etc. This is likely due to the complicated nature of accommodating respiratory motion, which requires a comprehensive understanding of robot and tissue dynamics, patient-dependent organ motion and characterization, and tracking and feedback of these systems. This is further complicated by the need for online registration between the mobile imaging transducer and the robot in USgHIFU [44]. This absence of available respiratory motion-compensated systems indicates a significant research need and a future direction for robotic development.

The authors would like to note that MRgHIFU systems provide an easier registration method as the patient and robot are registered to a common imaging frame (the MRI-coordinate imaging frame) as opposed to the imaging frame that moves in the USgHIFU case due to the mobile transducer. Further, MRI provides advanced thermometry techniques and high-contrast images for suitable HIFU monitoring. However, while MRgHIFU shows promise for the development of respiratory motion-compensated interventions, these systems tend to have less DoF and take a more rudimentary approach to targeting by not considering path-planning capabilities. Specifically, the majority of MRgHIFU systems focus on setpoint targeting (i.e., targeting a single lesion, which requires a minimum of 3 DoF), whereas USgHIFU enabled more advanced path-planning algorithms (for relatively stationary organs) to preserve healthy tissue. This is likely due to the preclusion of ferromagnetic materials in MR-guided interventions, which restricts actuator use to pneumatics, hydraulics, or piezoelectric actuators, where piezoelectric actuators typically do not operate synchronously with imaging as imaging quality is reduced [106,107]. Thus, this identifies an additional research need. While we hypothesize that future respiratory motion-compensated HIFU systems can benefit significantly from MR guidance, it is necessary to develop actuators, encoders, and sensors that can be used synchronously during MR imaging to enable future path-planning capabilities with synchronous imaging.

## Figures and Tables

**Table 1:** Overview of Ultrasound-guided HIFU Systems.

System	Subjects	Reported Treatments	DoF	Features	References
HIFUPlex Plus 1000	Animal	Extracorporeally reached tumors for small animals	2	Motion Compensation: No Compact benchtop form factor Electronically steered annular array that improves focal depth Imaging probe with various modes (B-mode, Doppler, and harmonic imaging) for treatment monitoring	[33,34,35]
VIFU 2000	Animal	Extracorporeally reached tumors	5	Motion Compensation: No Configurations for wet-type or dry-type Improved ablation depths with wet-type Reflected acoustic pressure monitoring Displays simulated treatment overlaid with B-mode image	[36,37,38,39,40]
PRO2008	Human	Myoma Adenomyosis Placental implantation Cesarean Scars Pancreatic Tumor Uterine Fibroids	4	Motion Compensation: No Guidance from B-mode and Doppler Ultrasound Phased Array HIFU Transducer for Beam Steering Integrated software that reduces uneven heating.	[41,42,43]
An et al.	Human	Extracorporeally reached tumors	4	Motion Compensation: No (but feasible) Optical tracker relates all coordinate frames Detailed coordinate transformation scheme Relatively inexpensive and uses readily available ultrasound components	[44]
Yonetsuji et al.	Human	Breast	4	Motion Compensation: No Rotary insonification decreases skin burn Rotary method creates more homogeneous ablation Portable form factor with cart	[45,46]
FUSBOT (BS;US;NS)	Human	Breast (BS) Urological Organs (US) Brain (NS)	5	Motion Compensation: No Multiple confocal HIFU transducers for more homogeneous ablation Adjusted acoustic power and path with multiple transducers Versions for different tumor treatments	[47,48,49,50,51]
ALPIUS 900	Human	Extracorporeally reached tumors Pancreas	5	Motion Compensation: Yes (with apnea) Positioning arm contains rotating confocal ultrasound probe in the center of the HIFU probe Electronically steered focus with multi-element HIFU transducer Minimal physician repositioning Pre-targeting and 3D modeling assisted treatment planning	[52]
Tang et al.	Human	Breast	5	Motion Compensation: No Parallel robot with confocal imaging and HIFU transducers Electrically steered focus with multi-element HIFU transducer Compact form factor positioned beneath patient table	[53]
Valvosoft	Human	Aortic valve	5	Motion Compensation: Yes Non-thermal high-focused ultrasound delivered transthoracically 2D echo-imaging monitoring Cardiac motion prediction incorporated in treatment plan Electrically steered focus with multi-element HIFU transducer	[19]
Groen et al.	Human	Peripheral arteries and surrounding tissues	6	Motion Compensation: No Automatic modulation of HIFU exposure based on echogenicity Single transmit imaging for HIFU delivery visualization Monitors skin interface Real-time target selection for ongoing procedure Beam steering with multi-element HIFU transducer	[54,55]
JC200	Human	Gynecological tumors Liver Bone Breast Pancreas	6	Motion Compensation: Yes Treatment of soft tissue tumors adjacent to blood vessels Interchangeable transducers Real-time dosage adjustment Margin between treated and untreated tissue as narrow as 10 cells wide	[56,57,58,59]
Chanel et al.	Human	Lungs Liver Respiratory organs	6	Motion Compensation: Yes Motion tracking with speckle tracking software Flexibility with region of motion tracking Fast processing time for moving target	[23]
FUTURA	Human	Extracorporeally reached tumors	6	Motion Compensation: Yes 6-DoF force sensors at end-effectors Software improves ablation targeting error with each sonication Fit with 3D ultrasound probe	[60,61]
Sonovein	Human	Varicose veins	6	Motion Compensation: No Robotic positioning capable of treatment near the femoral junction Laser on probe monitors acoustic coupling and focal distance	[20,62,63]

**Table 2:** Overview of Magnetic Resonance-Guided HIFU Systems.

System	Subjects	Reported Treatments	DoF	Features	References
Medsonic	Animal	Small Animal Tumors	2	Respiratory Motion Compensation: No MR-thermometry Relatively inexpensive 3D-printed components Compact form factor sits on MRI patient table Control of MR-compatible camera May be easily incorporated into the treatment table	[64]
Damianou et al.	Animal	Breast Liver Kidney Pancreas Bone	4	Motion Compensation: No MR-thermometry Adaptability for access to various regions in prone patient position Piezoelectric ultrasonic motors placed outside of the water container Compact form factor within MRI bore	[65]
Menikou et al.	Human	Bone	4	Motion Compensation: No Compact form factor sits on MRI patient table 6 treatment plan algorithms applicable Allows for more comfortable supine patient position	[66,67,68,69]
Yiallouras et al.	Human	Prostate	3	Motion Compensation: No Large range of motion (10 cm longitudinal × 7 cm elevation) 180° transducer rotation for minimal readjustment	[70].
Epaminonda et al.	Human	Gynecological Organs	2	Motion Compensation: No Imaging coil placed around the probe enclosure for optimal MRI scans Compact form factor sits on MRI patient table	[71]
TRANS-FUSIMO	Human	Liver Upper-abdominal organs	3	Motion Compensation: Yes Adaptive ablation parameters with movement Beam steering of focus with movement Beam forming Multi-baseline thermometry	[72,73,74,75]
Giannakou et al.	Human	Prostate	4	Motion Compensation: No MR-thermometry Compact form factor sits on MRI table and between patient legs Allows patient to lie in supine position Relatively inexpensive production with 3D-printed components	[76]
ExAblate 2000	Human	Uterine Fibroids Breast Liver Prostate	5	Motion Compensation: Yes (with apnea) Robotic system resides in the patient table Longest approved robotic-assisted HIFU system MR-thermometry	[77,78,79,80,81,82,83,84]
Sonalleve	Human	Uterine Fibroids	5	Motion Compensation: No Robotic system easily incorporated with the patient table Efficient concentric-circular ablation path Automatic adjustment of ablation with online thermal feedback Shorter ablation times MR-thermometry	[85,86,87,88]
Li et al.	Human	Brain	8	Motion Compensation: No Needle-based HIFU applicator Based on precise stereotactic frame positioning Active tracking coils on the needle insertion unit Minimally affected SNR from piezoelectric actuators Relatively inexpensive 3D-printed components	[89]

**Table 3:** Overview of Multi-Modal Image-Guided HIFU Therapy.

System	Subjects	Reported Treatments	DoF	Features	References
Focal One	Human	Prostate	4	Motion Compensation: No Real-time adjustment of ablation path Combines real-time ultrasound images with either MRI-data or 3D biopsy data	[90,91]
Innomotion	Human	Spine Liver Kidney Breast	5	Motion Compensation: Yes Form factor fits within MRI bore Orbital ring may reach extreme lateral tumor locations Wireless confocal imaging probe for real-time US imaging	[92,93]
Ablatherm	Human	Prostate	6	Motion Compensation: No System built into MRI patient table Overlays MR-image maps onto 3D ultrasound images in real-time MR-thermometry	[94,95]
Sonablate 500	Human	Prostate	6	Motion Compensation: No Monitors tissue destruction with pulse-echo signals per ablation site Monitors tissue temperature Displays MR-contours onto ultrasound images	[96,97,98]
